# Early detection of lung cancer in Czech high-risk asymptomatic individuals (ELEGANCE): A study protocol

**DOI:** 10.1097/MD.0000000000023878

**Published:** 2021-02-05

**Authors:** Lukas Lambert, Lenka Janouskova, Matej Novak, Bianka Bircakova, Zuzana Meckova, Jiri Votruba, Pavel Michalek, Andrea Burgetova

**Affiliations:** a Department of Radiology; b 1st Department of Tuberculosis and Respiratory Diseases; c Institute of Nuclear Medicine; d Department of Anesthesiology and Intensive Care, First Faculty of Medicine, Charles University and General University Hospital in Prague, Prague, Czech Republic.

**Keywords:** low-dose CT, lung cancer, Lung-RADS, nodule, pulmonary, screening, stage

## Abstract

**Background:**

Lung cancer screening in high-risk population increases the proportion of patients diagnosed at a resectable stage.

**Aims:**

To optimize the selection criteria and quality indicators for lung cancer screening by low-dose CT (LDCT) in the Czech population of high-risk individuals. To compare the influence of screening on the stage of lung cancer at the time of the diagnosis with the stage distribution in an unscreened population. To estimate the impact on life-years lost according to the stage-specific cancer survival and stage distribution in the screened population. To calculate the cost-effectiveness of the screening program.

**Methods:**

Based on the evidence from large national trials - the National Lung Screening Trial in the USA (NLST), the NELSON study, the recent recommendations of the Fleischner society, the American College of Radiology, and I-ELCAP action group, we developed a protocol for a single-arm prospective study in the Czech Republic for the screening of high-risk asymptomatic individuals. The study commenced in August 2020.

**Results:**

The inclusion criteria are: age 55 to 74 years; smoking: ≥30 pack-years; smoker or ex-smoker <15 years; performance status (0–1). The screening timepoints are at baseline and 1 year. The LDCT acquisition has a target CTDIvol ≤0.5mGy and effective dose ≤0.2mSv for a standard-size patient. The interpretation of findings is primarily based on nodule volumetry, volume doubling time (and related risk of malignancy). The management includes follow-up LDCT, contrast enhanced CT, PET/CT, tissue sampling. The primary outcome is the number of cancers detected at a resectable stage, secondary outcomes include the average cost per diagnosis of lung cancer, the number, cost, complications of secondary examinations, and the number of potentially important secondary findings.

**Conclusions:**

A study protocol for early detection of lung cancer in Czech high-risk asymptomatic individuals (ELEGANCE) study using LDCT has been described.

## 1 Introduction

The annual incidence rate of lung cancer in the Czech Republic is high, 86 and 43 cases per 100,000 men and women a year, respectively.^[1]^ With more than half of the cases diagnosed in stage IV, the relative 5-year survival is only 10% making it the most common cause of death among oncological diagnoses. Cigarette smoking is a well-documented cause of lung cancer and about 90% of lung cancers are directly caused by smoking. The relationship between the number of cigarettes smoked per day, the depth of inhalation, the age of the smoker, and the development of lung cancer have been documented.^[2]^

Vast resources have been dedicated to shifting the diagnosis of bronchogenic cancer to its early stages. Poor survival can be largely attributed to delayed diagnosis. Only small resectable stage I tumors offer favorable prognosis with 5-year survival rates of 70% to 90%.^[3]^

In the United States, where lung cancer screening is an established and recognized tool, smoking prevalence is only 15 percent. In the Czech Republic where every fourth adult and about 12% of primary school pupils are active smokers, there is no screening for lung cancer yet. Because of the unsatisfactory results of anti-smoking intervention programs and the fact that even more lung tumors are becoming diagnosed in former (non-active) smokers, secondary prevention by early detection of lung cancer by screening in a selected population is proposed.

Based on previous studies, including the National Lung Screening Trial in the USA (NLST),^[4]^ the NELSON study,^[5]^ and on the recent recommendations of the Fleischner society,^[6]^ the American College of Radiology^[7]^ and I-ELCAP action group,^[8]^ we developed a protocol for an optimization study in the Czech Republic for the screening of high-risk asymptomatic individuals by low-dose CT (LDCT).

## 2 Study protocol

The study was approved by the Ethics Committee of the General University Hospital in Prague (12/19 Grant AZV VES 2020 VFN). The study adheres to the principles of the Declaration of Helsinki. Written, informed consent to participate will be obtained from all participants. Patients may discontinue at any time. The participants are being recruited by family physicians, pneumologists, and self-referred by an advertising campaign. The study is designed as a single-arm prospective study conducted in an academic hospital. The study is registered under ClinicalTrials.gov ID: NCT04627350. Protocol modifications will be announced to the trial registry and the Ethics Committee.

### 2.1 Study aims

The aims of this project are:

1. to optimize selection criteria and quality indicators for the target population for lung cancer screening in the Czech population
2. to compare the influence of screening on the stage of lung cancer at the time of the diagnosis with the stage distribution in an unscreened population
3. to estimate the impact on life-years lost according to the stage-specific cancer survival and stage distribution in the screened population
4. to calculate the cost-effectiveness of the screening program
5. to assess the potential for opportunistic screening of non-communicable diseases.

### 2.2 Study objectives

#### 2.2.1 Primary objective

1) The number of cancers detected at a resectable stage (stage I) vs non-resectable stage (II-IV).

#### 2.2.2 Secondary objectives

1. The average cost per diagnosis of lung cancer at a resectable stage
2. Number, cost, complications of secondary examinations – follow-up LDCT, PET/CT, bronchoscopy, tissue sampling
3. Number of potentially important secondary findings (e.g., pulmonary fibrosis, aortic aneurysm, compression fracture and signs of osteoporosis,^[9,10]^ myocardial scar) previously unknown.

The study is designed as 2 screening rounds at a baseline and at 1 year, the estimated study duration is 4 years with 2 years of enrolment to baseline screening, 1 year for second round screening, and 1 year for follow-up of the second round. The study commenced in August 2020. Study data are entered to a secured enterprise database, which can be accessed by all investigators. The first author is responsible for data monitoring, integrity, and auditing on a monthly basis. Nominal data will be presented as numbers and percentages and will be analyzed using the Fisher test. Ordinal and continuous data will be reported as mean ± standard deviation or 95% confidence intervals. The study results will be published in a peer-reviewed journal. Authorship will be based on the ICMJE guidelines.

### 2.3 Sample size

In comparison with the Nelson study, where 2.1% scans were positive and 0.9% were cancers detected in the first round, we define a study population at greater risk and expect that the number of detected cancers would be greater. In the Nelson study 58.6% of cancers were detected at stage I compared to 13.5% in the control group. According to the Czech cancer registry, only 10.5% of lung cancers are detected in stage I.^[1]^ To compare these outcomes, the study would require 15 patients with detected cancer, about 1500 screened patients to achieve a power of 80% at a 0.05 significance level. For an expected adherence of 90% and the optimization of input values, we estimate the sample size at 2500 participants. This study is designed as observational, therefore it has no control arm and it has no ambition to assess the effect of lung screening on mortality such as in the NELSON trial with a sample size calculated between 17,300 and 27,900 participants.^[11]^

### 2.4 Inclusion and exclusion criteria

The pretest risk of lung cancer is dependent especially on the smoking duration, age, sex, family history of lung cancer.^[12,13]^ The inclusion criteria should define a population of participants with the highest risk of lung cancer who are capable of undergoing curative treatment if cancer is found. The recommended threshold by the UK Lung Cancer Screening Trial was based on the prediction result of at least 5% risk of developing lung cancer in the following 5 years.^[12]^

**Figure 1:**
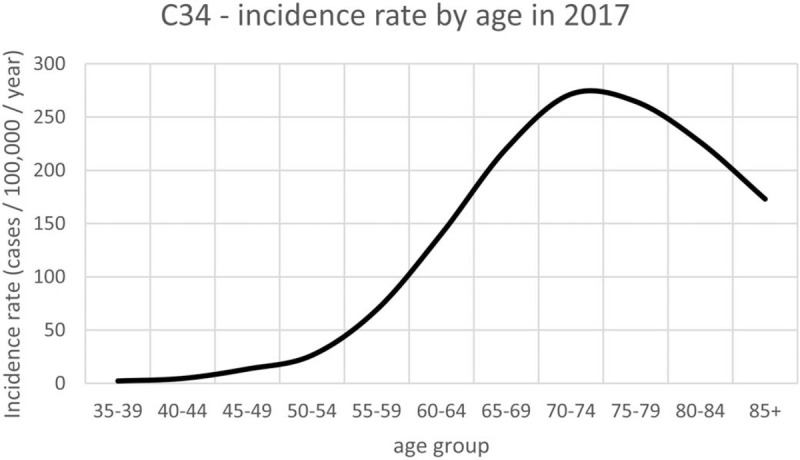
Incidence rate of C34 (Malignant neoplasm of bronchus and lung) by age in 2017 in the Czech Republic. Data from the National Oncology Registry.^[1]^

The highest incidence of lung cancer in the Czech Republic is between 60 to 79 years (Fig. 1).^[1]^ The lung screening trials with the highest sample sizes included patients aged approximately between 55 and 75 years of age.^[14]^ The CHEST Guideline and Expert Panel Report recommend the optimal screening 55 to 77 years.^[15]^ The U.S. Preventive Services Task Force recommends annual screening up to 80 years of age, while the Centers for Medicare & Medicaid Services (CMS) end coverage at the age of 77, corresponding to the oldest age at the time of the final annual screen in the NLST. The optimal age span for screening was set to 55 to 74 years to precede the diagnosis of advanced lung cancer and to take into account a shorter life expectancy in the Czech Republic. The male gender carries a higher risk of lung cancer by a factor of 1.5 to 2.^[16]^ This fact is related to a smaller proportion of women, who are heavy smokers and can therefore be equalized by the pack-years inclusion criterion.

The best predictor of lung cancer is smoking duration and intensity. It is expressed as the number of cigarettes per day for a given number of years (“pack years”, 1 pack = 20 cigarettes, 1 pack-year = 20 cigarettes daily for 1 year). Various thresholds have been used with a range from 15 pack-years up to 30 pack-years.^[14,15,17]^ Greater risk means better efficacy of screening. The pause from becoming a non-smoker was determined between 10 and 15 years in various studies, for it is known that after this period the risk of developing lung cancer decreases. The recommendations of the CHEST Guideline and Expert Panel Report recommend a threshold of 30 pack-years and <15 years from the cessation of smoking.^[15,18]^

Further risk factors include obstruction on spirometry (FEV-1 below 70% has OR 2.9), which is a criterion that would be difficult to implement.^[19]^ Moreover, patients with greater obstruction have lower vital capacity limits and higher perioperative risk if resection would be considered.

Patients who are not amenable to curative treatment due to their poor performance status are unlikely to benefit from the early detection of lung cancer. The performance of the patients can be assessed by their ability to undergo physical exertion such as climbing the stairs. In the DLCST trial, the threshold was the ability to climb 36 steps without pause.^[20]^ We define good performance status as the ability to climb stairs at least 1 floor without any difficulty or pause.

#### 2.4.1 Inclusion criteria

- Age 55 to 74 years
- Smoking: ≥30 pack-years, smoker or ex-smoker <15 years
- Performance status (0–1) – can climb at least 1 floor without any difficulty or pause

#### 2.4.2 Exclusion criteria

- Body weight above 140 kg
- Malignant disease within the last 10 years (except non-melanoma skin cancer).
- Chest CT less than 1 year ago, chest x-ray ≤6 months ago
- Clinical signs suspicious of lung cancer (weight loss, new cough, hemoptysis)
- Recent (≤2 months) bronchopneumonia, pneumonia

### 2.5 Screening interval

The selection of the optimal screening interval, which can be obtained by processing the NELSON study data, is important as well. It appears that the extension of the screening interval (in the NELSON trial it was 1 year, 2 years, and 2.5 years) brings a stationary incidence of diagnosed tumors to a screening round (0.8%), but worsening of the average stage of newly diagnosed tumors in the last round of screening (i.e., 2.5 years).^[21]^ Thus, the 2.5-year interval was too long. The screening interval was set at 12 months.

### 2.6 Low-dose CT (LDCT)

The CT settings for screening protocols varied between 80 and 140 kVp with a minimum of 20 mAs tube time-current product.^[15]^ The reported effective doses ranged from <0.4 mSv in lean patients to 2mSv. More recent trials adopted automated kV selection and mAs modulation based on the attenuation profile of the patient. Sufficient image quality and diagnostic performance of ultra-low-dose protocols (<0.2 mSv) for the detection of pulmonary nodules and even ground-glass lesions with the use of model-based iterative reconstruction technique has been confirmed in phantom and clinical studies.^[22,23]^ For a standard-size patient, this corresponds to a CTDIvol <0.6 mGy for a 25 cm scan.^[13,18]^

#### 2.6.1 CT acquisition protocol

- ≥64 slice scanner
- Axial and longitudinal current modulation
- Iterative reconstruction – hybrid or model-based
- Tube voltage: 80–120 kV – automated selection (auto kV)
- Tube current: ref. 10 mAs (automated modulation) – target CTDIvol≤0.6 mGy (standard-size patient), effective dose ≤0.2 mSv
- Extent: lung apices – costophrenic angles
- Command: “Take a deep breath and hold it”.

#### 2.6.2 Image reconstruction

Isotropic (≤1 mm), soft tissue (Body kernels [B, Bf, Br, BI] 20–42 for Siemens or equivalent), contiguous or overlapped, axial plane, iterative reconstruction (hybrid, model-based), and

- 5 mm axial contiguous
- 5 mm coronal contiguous
- 5 mm sagittal contiguous

≤1 mm volumetric isotropic (≤1 mm), soft tissue (Body kernels [B, Bf, Br, BI] 50–70 for Siemens or equivalent), contiguous or overlapped, axial plane, iterative reconstruction (hybrid, model-based) optional, and

- 1.5 mm axial contiguous

### 2.7 Interpretation of LDCT

The interpretation of CT findings is based on the probability that a lesion harbors malignity and how fast it can develop beyond the localized stage. Several academic groups including the Fleischner society, the British thoracic society, the American College of Chest Physicians have proposed guidelines on the management of pulmonary nodules. They derive the risk and the need for further management from the imaging features of the nodule (size or volume, spiculation, upper lobe location, perifissural location), the lungs (emphysema), and the pretest probability of malignancy (age, family history of malignancy, obstruction).^[24,25]^

The best predictor of malignancy of a solid nodule is its size and growth rate.^[26]^ From a mean diameter of 6 mm it starts to increase steadily.^[27]^ The prevalence of malignancy in nodule <5 mm is extremely low, about half a percent.^[28]^ In the NELSON trial, the risk of malignancy in subjects with nodules <100 mm^3^ was similar to those without nodules.^[29]^ Based on these data, the lower size threshold for nodules that require follow up was increased to 6 mm or 100 mm^3^ in the Fleischner society guidelines^[6]^ and 5 mm or 80 mm^3^ in the BTS guidelines.^[30]^

The NELSON study showed that small nodules (<100 mm^3^) are not predictive for lung cancer and that nodules ≥300 mm^3^ or ≥10 mm require timely attention.^[29]^ Malignant nodules showed an exponential growth and nodule doubling time with a threshold of 600 days was suggested as the most optimal for nodules sized between 100 and 300 mm^3^.^[31]^

There is little information about the behavior of subsolid nodules. Ground-glass nodules show very slow growth, beyond the 600 days VDT threshold, and are safe to follow-up on an annual basis.^[6]^ In part-solid nodules, the size of its solid component and its growth are predictive of lung cancer.

The annual incident rates of new nodules (ELCAP, I-ELCAP, PLuSS, and Mayo trials) are reported between 3.4 and 13%. These new nodules are logically fast-growing and the data we have available show that the probability of malignancy in such a newly emerging nodule is 1.6% to 7.5%. Therefore, it may be necessary to choose a lower volume limit for these new nodules than for the first round of screening.^[31]^ The models that aim to distinguish between malignant and benign nodules include the model of McWilliams and the American College of Radiology model, which in 2014 published its Lung-RADS assessment criteria, which were updated in 2019 (Lung-RADS 1.1).^[7]^ The Lung-RADS model is the most cited as it is used in virtually all U.S. screening centers. It includes 5 categories that determine the type of lesion and its subsequent management based on the type of the lesion, its size, and behavior.

About half of the asymptomatic high-risk individuals undergoing screening by LDCT present with more than 1 nodule. At baseline, in the NELSON trial, where the management was based on the largest or most suspicious nodule, malignancy was detected mostly in the largest nodule (97%).^[31]^ However, in the PanCan study, one-fifth of the positive individuals were diagnosed with cancer in a lesion that was not the largest.^[27]^

### 2.8 Evaluation and management of pulmonary nodules

In this study, the nodules will be assessed in Intellispace Portal (current version 10) Lung analysis package. This package performs automated detection of nodules and automatic segmentation of their volume with manual adjustment by the radiologist, where necessary. Nodules that would escape automatic detection will be segmented manually (lung window) - the agreement between manual and automated segmentation is reported to be excellent.^[24]^ In lesions, where volume segmentation would be difficult or unreliable (e.g., perihilar), the effective diameter will be used (the average of 2 maximal perpendicular diameters), with the exception of broad-based subpleural lesions, where short diameter will be used instead. In non-solid nodules, the ground-glass part will be measured by effective diameter regardless of segmentation.

The management will be based on the largest or most suspicious nodule. Nodules 70 mm^3^ and larger will be recorded and their doubling time calculated if they would be found in the previous scan, wherever available. A nodule is by definition a rounded (spherical, oval) circumscribed focus of abnormal tissue.

The proposed management protocol uses the recursive definition of nodules (with regard to their growth). It is based on the recommendations of the American College of Radiology (ACR), The Fleischner society guidelines, and results of the NELSON trial.^[32,33]^ The primary assessment of risk (that a lesion harbors malignity) is based on volumetry and doubling time (Tables 1–3). For solid nodules, the volume threshold is 100 mm^3^ (70 mm^3^ for new nodules), the VDT threshold is 600 days. Subsolid nodules are rare (0.7%) and the risk of malignancy or premalignancy is low (6%) unless a new solid component appears.^[34,35]^ The data on the management of sub-solid nodules are scarce which is reflected in the diversity of recommendations across different guidelines.^[36]^ The update of the Lung-RADS model in 2019 increased the lower boundary for purely ground-glass nodules that require further management to 30 mm.^[7]^

**Table 1:** Management of solid nodules.

SOLID NODULES				
Description	Findings	Management	Risk	Estimated prevalence
1 - Benign appearance or behavior	• normal or benign findings^∗^	→1 year round	<1%	90%
	• perifissural nodule <9.8 mm (<500 mm^3^)^∗∗^			
	• <100 mm^3^ (<5.8 mm)			
	• new <70 mm^3^ (<5.1 mm)			
	• nodules with VDT >600 days			
	• benign histology, PET/CT negative			
2 - Low risk	• ≥100 to <300 mm^3^ (≥5.8 to <8.3 mm)	• 5 month LDCT	1%–6%	8%
	• new ≥70 to <100 mm^3^ (≥5.1 mm to <5.8 mm)			
3 - Intermediate risk	• ≥300 to <750 mm^3^ (≥8.3 to <11.2 mm)	• 3 month LDCT	9%–11%	2%
	• growing ≥100 to <300 mm^3^ (≥5.8 to <8.3 mm)	• optional *PET/CT* if ≥300 mm^3^ (≥8.3 mm)		
	• new ≥5.8 to <8.3 mm (≥100 to <300 mm^3^)			
	• endobronchial nodule			
4 - High risk	• ≥11.3 mm (≥750 mm^3^)	• PET/CT	19%–26%	1%
	• new or growing, and ≥ 8.3 mm (≥300 mm^3^)	• Tissue sampling^∗∗∗^		
	• other features suggesting malignancy (spiculation)	• Contrast-enhanced CT of thorax		
		• Equivocal appearance of a benign finding: 1–2 month LDCT		

**Table 2:** Management of part-solid nodules.

PART-SOLID NODULES		
Description	Findings	Management
1 - Benign appearance or behavior	• <8.3 mm total diameter (<300 mm^3^)	→ 1 year round
	• solid part VDT>600d	
	• benign histology, PET/CT negative	
2 - Low risk	• ≥8.3 mm total diameter (≥ 300 mm^3^) and solid part <100 mm^3^ (<5.8 mm)	• 5 month LDCT
	• new ≥5.1 to <8.3 mm total diameter (≥70 to >300 mm^3^)	
3 - Intermediate risk	• ≥8.3 mm (≥300 mm^3^) and	• 3 month LDCT
	a] solid part ≥100 to <300 mm^3^ (≥5.8 to <8.3 mm)	• optional PET/CT if solid part ≥300 mm^3^
	b] new or growing solid part <70 mm^3^ (<5.1 mm)	(≥ 8.3 mm)
4 - High risk	• solid part ≥300 mm^3^ (≥8.3 mm)	• PET/CT
	• new or growing solid component >70 mm^3^ (>5.1 mm)	• Tissue sampling^∗^
	• other features suggesting malignancy	• Contrast-enhanced CT of thorax
		• Equivocal appearance of a benign finding:
		1–2 month LDCT

**Table 3:** Management of non-solid nodules.

NON-SOLID NODULES		
Description	Findings	Management
1 - Benign appearance or behavior	• <30 mm diameter (<14000 mm^3^)	→ 1 year round
	• ≥30 mm diameter (≥14000 mm^3^) and stable	
2 - Low risk	• <30 mm diameter (≥14000 mm^3^) and growing	• 5 month LDCT
	• ≥30 mm diameter (≥ 14000 mm^3^)	
3 - Intermediate risk	–	–
4 - High risk	• ≥30 mm diameter (≥14000 mm^3^) and growing >1.5 mm / VDT<600 days	• PET/CT
	• other features suggesting malignancy	• Tissue sampling^∗^
		• Equivocal appearance of a benign
		finding: 1–2 month LDCT

Volume doubling time (VDT) based on volume (V) measurements:

VDT = [ln2 ∗ (T1 – T0)] / [ln (V1/V0)]

Volume doubling time based on mean diameter (D) measurements:

VDT = [ln2 ∗ (T1 – T0)] / [3 ∗ ln (D1/D0)].

Where T1 and T0 are timepoints of the measurement.

A positive screening test is defined as a high risk finding or intermediate risk finding where verification (PET/CT, tissue sampling, contrast-enhanced CT) was requested. An indeterminate test is defined as low and intermediate risk finding, further managed by LDCT. A negative test (benign appearance or behavior) does not mean that an individual does not have lung cancer.

Secondary findings will be reported as incidental findings and categorized according to their expected clinical significance as shown in Table 4.

**Table 4:** Classification of secondary findings.

SECONDARY FINDINGS	
	Description
1	Normal exam with regard to patient's age, benign variants, clinically unimportant findings
	- e.g., lobus v. azygos, degenerative spine disease, vertebral hemangioma
2	Potentially important findings
	- e.g., pleural effusion, pericardial effusion (>10mm), pulmonary fibrosis, mediastinal lymphadenopathy, pneumonia, emphysema, vertebral compression, aortic aneurysm

## Author contributions

**Conceptualization:** Lukas Lambert, Lenka Janouskova, Jiri Votruba, Andrea Burgetova.

**Data curation**: Lukas Lambert, Lenka Janouskova.

**Formal analysis:** Lukas Lambert.

**Funding acquisition**: Lukas Lambert, Pavel Michalek, Andrea Burgetova.

**Investigation**: Lukas Lambert, Lenka Janouskova, Matej Novak, Bianka Bircakova, Zuzana Meckova, Jiri Votruba.

**Methodology**: Lukas Lambert, Andrea Burgetova, Jiri Votruba.

**Project administration:** Lukas Lambert.

**Resources**: Lukas Lambert, Pavel Michalek, Jiri Votruba.

**Software**: Lukas Lambert.

**Supervision**: Lukas Lambert.

**Validation**: Lukas Lambert.

**Visualization**: Lukas Lambert.

**Writing – original draft:** Lukas Lambert.

**Writing – review & editing**: Lukas Lambert, Lenka Janouskova, Matej Novak, Bianka Bircakova, Zuzana Meckova, Jiri Votruba, Pavel Michalek, Andrea Burgetova.
